# Type A Aortic Dissection: From Diagnosis to Cardiac Rehabilitation

**DOI:** 10.3390/jcm15072749

**Published:** 2026-04-05

**Authors:** Monica Loguercio, Maria Grazia Romeo, Buket Akinci, Cristina Andreea Adam, Irfan Ullah, Marta Supervía, Giancarlo Trimarchi, Natalia Świątoniowska-Lonc, Federica Fogacci, Francesco Perone

**Affiliations:** 1 Cardiovascular Rehabilitation Unit, ASST Crema, Santa Marta Hospital, 26027 Rivolta D’Adda, Italy; msloguercio@gmail.com; 2 Department of Advanced Biomedical Science, Cardiac Surgery, University “Federico II”, 80131 Naples, Italy; mariagrazia2791@gmail.com; 3 Department of Physiotherapy and Rehabilitation, Faculty of Health Sciences, Biruni University, 34010 Istanbul, Turkey; barbuket@hotmail.com; 4 Biruni University Research Center (B@MER), Biruni University, 34010 Istanbul, Turkey; 5 Department of Medical Specialties I, “Grigore T. Popa” University of Medicine and Pharmacy, University Street No. 16, 700115 Iasi, Romania; adam.cristina93@gmail.com; 6 Clinical Rehabilitation Hospital, Cardiovascular Rehabilitation Clinic, Pantelimon Halipa Street No. 14, 700661 Iasi, Romania; 7 University Hospitals Harrington Heart & Vascular Institute, Cleveland, OH 44106, USA; irfanullahecp2@gmail.com; 8 Department of Physical Medicine and Rehabilitation, Gregorio Marañón General University Hospital, Gregorio Marañón Health Research Institute, 28007 Madrid, Spain; msuperviapola@gmail.com; 9 Rehabilitation and Physiotherapy Department, Complutense University School of Medicine, 28040 Madrid, Spain; 10 Division of Preventive Cardiology, Department of Cardiovascular Medicine, Mayo Clinic, Rochester, MN 55905, USA; 11 Interdisciplinary Center for Health Sciences, Scuola Superiore Sant’Anna, 56127 Pisa, Italy; giancarlo.trimarchi18@gmail.com; 12 Fondazione Toscana G. Monasterio, Ospedale del Cuore G. Pasquinucci, 54100 Massa, Italy; 13 Department of Cardiology, Center for Heart Diseases, 4th Military Hospital, 50-981 Wroclaw, Poland; natalia.swiat@o2.pl; 14 Hypertension and Cardiovascular Risk Research Center, Medical and Surgical Sciences Department, Alma Mater Studiorum University of Bologna, 40130 Bologna, Italy; federicafogacci@gmail.com; 15 Cardiac Rehabilitation Unit, Rehabilitation Clinic “Villa delle Magnolie”, 81020 Castel Morrone, Caserta, Italy

**Keywords:** cardiac rehabilitation, Type A aortic dissection, transthoracic echocardiography, transesophageal echocardiography, cardiovascular computed tomography, aerobic exercise training, resistance exercise training

## Abstract

Acute type A aortic dissection is a life-threatening condition requiring emergency surgery and complex postoperative management. Although survival rates have improved, many patients experience long-term functional impairments, reduced quality of life, and an elevated risk of complications. Despite strong evidence supporting cardiac rehabilitation in other cardiovascular populations, structured programs remain underutilized in patients with surgically resolved acute type A aortic dissection. Exercise-based cardiac rehabilitation appears feasible and can be delivered safely in carefully selected patients when appropriately adapted to individual needs and conducted under close supervision. Postoperative patients are often physically deconditioned, prone to hospital-acquired disability, and may misjudge exercise intensity. Therefore, individualized exercise prescription, guided by exercise testing when available, is important to support safe training thresholds. Early and gradual introduction of physical activity may help prevent complications associated with immobility, support blood pressure control, and contribute to improvements in functional capacity. However, training volume should be purposefully lower than in conventional program settings to reduce hemodynamic stress. Education on safe exercise parameters and self-monitoring plays a central role in enabling long-term adherence and promoting patient autonomy. Cardiac rehabilitation programs should incorporate dietary, nutritional, and psychological support. Although evidence specific to this patient population remains limited, available data suggest the feasibility and potential benefits of cardiac rehabilitation when delivered with appropriate precautions. Our review underscores the need for a tailored, multidisciplinary CR approach aimed at enhancing physical recovery, supporting cardiovascular stability, and improving overall quality of life in patients following surgery. Further research is required to define optimal program protocols.

## 1. Introduction

Aortic dissection is a cardiac emergency with a high mortality rate of up to 90% [1] in the absence of prompt diagnosis and rapid surgical management. Acute Type A aortic dissection (ATAAD) may involve any portion of the ascending aorta, frequently involving the proximal segments of the left subclavian artery [2]. Despite technological advances and the widespread use of cardiac computed tomography in patients with a suggestive clinical picture [3], epidemiologic studies report an initial mortality of 40%, which subsequently increases progressively by 1% per hour. The first case of aortic dissection was reported during the autopsy of King George II in 1760 [4]. Since then, the development of diagnostic methods has been associated with improved survival rates, which remain around 50% on the third day for cases not surgically treated [5]. In the case of 30-day mortality no sex differences were reported [6]. The estimated incidence is 2–4 cases per 100,000/year [7]. Type A dissection is twice as common in men, frequently occurring in the sixth decade of life. Risk factors are varied, with hypertension (around 75% of cases) [7], Marfan’s syndrome or other connective tissue disorders, and pre-existing aneurysms being the most frequently implicated entities, also associated with a younger age at onset [8]. Not only comorbidities are associated with type A aortic dissection, cardiac catheterization and cardiac surgery are also implicated.

Recent epidemiologic studies have highlighted additional factors such as pregnancy (4-fold increased risk) [9], bicuspid valve (8-fold increased risk) [10,11], familial thoracic aortic aneurysm in dissection syndrome [12] or cocaine/methamphetamine use. In addition to the aforementioned entities, weight lifting or traumatic cardiac deceleration or torsion in association with aortic coarctation are predisposing conditions leading to the occurrence of dissection [12]. The clinical picture is diverse considering the emerging arteries. The propagation of proximal dissection is often accompanied by acute aortic regurgitation, tamponade or myocardial infarction, while the extension to the emerging branches leads to the appearance of neurologic phenomena or the clinical picture associated with mesenteric or renal ischemia such as acute limb ischemia [13,14,15]. Management is centered on life-saving surgery, leading to a significant reduction in the risk of death at 30 days from 90% to 20% [16]. Along with intervention, the control and treatment of predisposing factors have long-term therapeutic and prognostic implications in this category of patients [17,18]. In addition to acute treatment, cardiac rehabilitation (CR) has the potential to contribute to functional recovery and to be associated with improved quality of life [7]. Evidence supporting these potential benefits derives mainly from small clinical trials and observational studies, while high-quality randomized controlled data remain limited. This limitation underscores the rationale for encouraging referral of patients with aortic dissection to specialized centers with expertise in tailored cardiac rehabilitation programs [19]. The available observational and prospective studies, together with narrative reviews, suggest that cardiac rehabilitation in this population may be feasible and generally well tolerated; however, these findings should be interpreted with caution and reinforce the need for larger, methodologically robust studies [20,21,22].

According to available expert reviews and commentaries informed by registry data, physical training in these patients has been associated with improvements in functional capacity, blood pressure control, cardiovascular risk profile, muscular endurance, and tolerance to parietal stress [23,24]. Taking into account the limited recommendations provided by current clinical guidelines of the relevant societies, our article aims highlight the potential importance and benefits of cardiac rehabilitation in patients with ATAAD surgically resolved. Multidisciplinary approach involving both physical training (adapted to each phase of the program) [25], management of associated factors and psychosocial counseling has an important prognostic role in the long term, decreasing morbidity and mortality of these patients.

## 2. Type A Aortic Dissection: Diagnosis and Treatment

Aortic dissection (AD) is a surgical emergency caused by an intimal tear that allows blood to enter the tunica media, leading to separation of vessel wall layers [26]. AD belongs to the group of acute aortic syndromes, which include penetrating aortic ulcer, intramural hematoma [27], aortitis, and endoluminal thrombosis. These are life-threatening conditions that share similar clinical features [28].

According to the Stanford classification, ATAAD involves the ascending aorta (proximal to the brachiocephalic artery), regardless of the site of the primary tear. It can also be classified as DeBakey type I/type II, depending on the distal extent of dissection [2]. The most recent classifications approved by the European Association for Cardio-Thoracic Surgery (EACTS) characterize AD based on the location of the primary tear, the extent of EAthe false lumen, and the presence of malperfusion. This is referred to as the TEM (Type-Entry-Malperfusion) classification in the EACTS/STS 2024 guidelines [29]. The goal of this new system is to provide a standardized nomenclature for AD to guide diagnosis and treatment [29].

The symptoms of ATAAD are variable and nonspecific, making differential diagnosis with other acute cardiac conditions challenging. In more than 90% of cases, the initial symptom is sudden chest pain. Depending on its location and extent, ATAAD may also present with acute stroke, paraplegia, abdominal pain, renal failure, or lower limb ischemia. From a hemodynamic perspective, patients with ATAAD typically present normotensive/hypotensive, often with tachycardia. Hypotension may indicate aortic rupture, cardiac tamponade, myocardial infarction, or acute heart failure secondary to aortic regurgitation (AR) [30]. Physical examination may reveal widened pulse pressure or an AR murmur, and up to 30% of patients may exhibit a pulse deficit. Left-sided pleural effusion is common in ATAAD, whereas acute hemothorax suggests impending rupture [31].

Medical therapy is critical for initial stabilization and should aim to reduce excessive shear stress on the dissected layers, thereby limiting propagation of the false lumen and preventing worsening of complications such as severe AR, hypotension, and cardiac tamponade [32]. Management of blood pressure and heart rate may include intravenous β-blockers such as esmolol, metoprolol, or labetalol, and intravenous vasodilators such as sodium nitroprusside, nicardipine, or clevidipine, but the latter should always be combined with a β-blocker to prevent reflex tachycardia. Intravenous morphine is the analgesic of choice, providing both pain relief and venodilation.

Diagnostic evaluation includes chest X-ray (CXR), electrocardiogram (ECG), and laboratory tests to rule out other causes of chest pain [33]. Measurement of D-dimer may be useful in emergency settings; Yao et al. demonstrated that a D-dimer level > 500 ng/mL increases the likelihood of identifying suspected acute AD [34]. CXR helps assess aortic size and contour abnormalities (such as the loss of aortic knob), identify pleural effusion or pneumothorax, and provide information on lung structure and the mediastinum in patients undergoing cardiac surgery [35]. ECG may be useful for excluding other causes of chest pain. Transthoracic echocardiography (TTE) represents a valuable initial bedside tool, particularly in hemodynamically unstable patients or when a rapid assessment of complications is required. It provides preoperative anatomic information on the ascending aorta, aortic arch, the presence and location of intimal flaps, valve involvement, degree of AR, left ventricular function, and cardiac tamponade [36]. Transesophageal echocardiography is essential intraoperatively to assess aortic valve anatomy and function and to guide both surgical and endovascular repair by evaluating true and false lumens before and after intervention. However, cardiovascular computed tomography angiography (CTA), preferably ECG-gated—owing to its rapid acquisition, wide availability, and high reproducibility—is the most accurate modality for diagnosis, prognostic assessment, and surgical planning in ATAAD and it is considered the first-line imaging modality in most centers for the definitive diagnosis of acute aortic dissection in hemodynamically stable patients. ECG-gated CTA of the entire aorta is regarded as the reference imaging modality for suspected aortic dissection, owing to its very high sensitivity and specificity, typically reported in the high 90% range in contemporary studies, and should be performed as soon as possible to localize the entry tear, assess the extent of dissection and malperfusion [37], and enable a tailored surgical approach.

**Figure 1:**
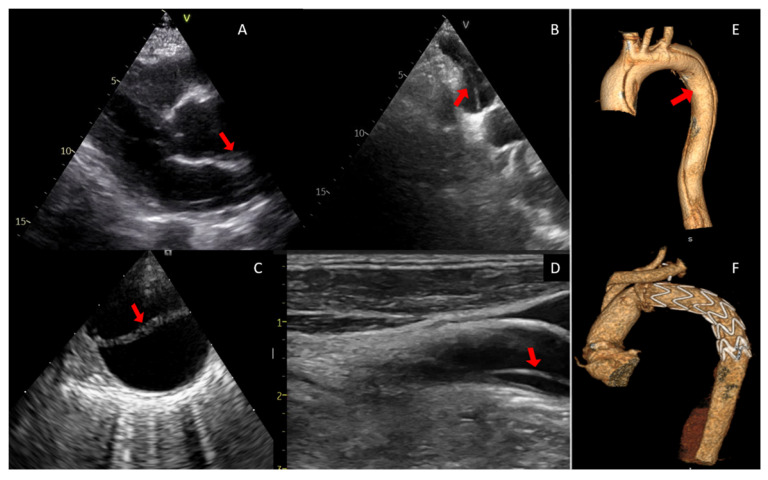
(**A**) TTE, parasternal long-axis view showing the presence of an intimal flap in the ascending aorta (arrow) and the correct anatomical conformation of the aortic root. (**B**) Intraoperative suprasternal section of the aortic arch showing an intimal dissection flap and the passage of the guidewire in the aortic arch (arrow) for the correct hybrid (endovascular/surgical) positioning of the prosthesis in the true lumen. (**C**) Intraoperative TEE, in short axis view of the aorta, revealing a double lumen in the descending aorta and the presence of the intimal flap (arrow). (**D**) Color Doppler ultrasound of the SAV showing evidence of dissection extending to the carotid artery (arrow). (**E**) CTA-3D reconstruction of an aortic dissection extending from the aortic root to the abdominal aorta, involving the SAV; the arrow indicates the extension of the true lumen in the small curvature of the aortic arch. (**F**) 3D CTA reconstruction after surgical repair of the ascending aorta and aortic arch using the FET technique and hybrid prosthesis, necessary to assess the correct functioning and positioning of the prosthesis.

Early surgical repair—ideally within the first 48 h after symptom onset—is lifesaving. According to data from the International Registry of Acute Aortic Dissection (IRAD), patients treated medically have a mortality rate of 23.7% (0.5% per hour), compared with 4.4% (0.09% per hour) in those undergoing surgery [38]. Surgery is the standard of care for this condition, although exceptions may exist, such as patients at extreme risk who are not candidates for surgery, or type B dissections, which may be managed endovascularly [39,40]. The surgical strategy depends on the extent of involvement of the aortic root; procedures may include aortic valve replacement or valve-sparing techniques. When the dissection extends to the supra-aortic vessels (SAV) and the aortic arch, the preferred approach is the frozen elephant trunk (FET) technique for arch replacement and SAV reimplantation [29] (Figure 1). Mortality risk depends on multiple factors and ranges from 5% to 75% [41]. According to the GERAADA (German Registry of Acute Aortic Dissection Type A) score, key predictors of 30-day postoperative mortality include age, sex, preoperative resuscitation, prior cardiac surgery, intubation or catecholamine use at admission, AR, malperfusion, neurological status, and extent of dissection [42].

## 3. Management After Cardiac Surgery (Post-Operative Period)

While prompt diagnosis and acute surgical management are essential to ensure survival in patients with ATAAD, they also mark the beginning of a complex postoperative phase. Clinical features identified during the acute stage—such as surgical technique, residual aortic disease, and early complications—have important implications for subsequent management and recovery. Optimal postoperative treatment requires careful monitoring of hemodynamic parameters and respiratory function. In the immediate postoperative period, optimization of inotropic support should be undertaken as needed, and, following extubation, individualized medical therapy should be initiated according to the patient’s clinical profile [43].

Imaging plays a crucial role not only intraoperatively, but also in the early postoperative phase, and throughout long-term follow-up, and includes assessment by TTE, CXR, and CTA. Postoperative TTE enables monitoring of ventricular function, evaluation of proper positioning of the prosthetic graft and/or aortic prosthesis, assessment of aortic valve function, and estimation of aortic remodeling and hemodynamic status, including monitoring of volume overload or depletion [44]. TTE is also valuable for detecting post-cardiotomy pericardial effusion and for pulmonary evaluation, allowing estimation of pleural effusion, pneumothorax, or regions of ventilation impairment. Following ATAAD surgery, patients may experience complications related to anesthesia, mechanical ventilation, prolonged cardiopulmonary bypass relative to other cardiac procedures, and postoperative pain from surgical wounds. These factors can lead to loss of cognitive and physical function, muscle wasting, and reduced pulmonary capacity. Physical inactivity during the recovery phase often results in prolonged intensive care unit (ICU) stays, which are themselves associated with worse clinical outcomes.

CR is a medically supervised, guideline-recommended Class IA cornerstone of secondary prevention for patients with cardiovascular disease [45], improving functional capacity, endothelial function, musculoskeletal performance, and quality of life and reducing mortality and hospital readmissions [46]. Within this framework, early mobilization is essential to mitigate ICU-acquired weakness. To be effective, it must be tailored to the individual patient’s functional capacity and performed under close monitoring of vital parameters, as patients in intensive care may experience abrupt hemodynamic changes [47]. Mobilization should begin with passive techniques such as stretching, splinting, passive joint movements, and neuromuscular electrical stimulation (NMES), alongside inspiratory muscle training [48]. Passive mobilization and NMES are typically implemented in intubated patients on mechanical ventilation, high-risk patients with hemodynamic instability, or those with impaired consciousness. In contrast, assisted joint exercises, inspiratory muscle training, and resistance activities are indicated for patients with adequate cognitive and physical status. Several preliminary studies have demonstrated superior outcomes when activity is initiated early [49]. As soon as chest drains are removed, inspiratory muscle training is recommended to enhance pulmonary function, facilitate expectoration and bronchial secretions clearance, and restore lung capacity following mechanical ventilation [50]. To minimize adverse events, a multidisciplinary approach involving a team of specialists and a personalized rehabilitation program tailored to the patient’s functional status are essential [51]. Upon discharge from the ICU and transfer to the ward, active mobilization and ambulation should be progressively encouraged, beginning with short walks of a few minutes that are gradually increased in duration and intensity. Early rehabilitation programs must be individualized, taking into account each patient’s functional abilities and recovery trajectory. Although several position papers and observational studies suggest that cardiac rehabilitation after aortic surgery can be safe in carefully selected patients, clear and standardized criteria for postoperative risk stratification and CR referral are still lacking. This underscores the need for pragmatic tools that translate the available evidence into clinically usable frameworks, while awaiting prospective validation. Accordingly, Table 1 presents a proposed checklist for aorta-specific postoperative risk stratification and cardiac rehabilitation planning. This checklist is intended as an evidence-aligned clinical support tool rather than validated guideline-level criteria. Individualized clinical assessment remains essential, and further prospective studies are needed to establish standardized risk stratification and rehabilitation pathways after aortic surgery.

## 4. Cardiac Rehabilitation

Although advances in diagnosis and treatment have improved survival in ATAAD, mid-term outcomes remain poor due to ongoing aortic dilation and persistent wall stress [52]. Exercise training, a cornerstone of CR, is well recognized for its broad benefits across various cardiovascular diseases, including improved blood pressure control, better risk factor management, slowed progression of atherosclerosis, enhanced quality of life, and improved long-term outcomes [53]. However, its role in aortic dissection remains uncertain. Concerns persist about the potential for exercise-induced blood pressure spikes to increase aortic wall stress, yet prolonged inactivity may also be harmful, contributing to vascular dysfunction and increased cardiovascular risk [19].

Structured exercise programs are rarely implemented after ATAAD repair reflecting both the limited available evidence and the absence of clear guidance regarding optimal timing, intensity, and modalities of rehabilitation [53]. Although the European Society of Cardiology encourages leisure-time physical activity in patients with aortic disease, favoring dynamic over static exercise, it does not specifically define the role of CR in this population. Evidence supporting cardiac rehabilitation after ATAAD repair is mainly derived from small, observational, and often single-center studies, with heterogeneous populations, interventions, and outcome measures. Sample sizes are limited, follow-up is variable, and selection bias cannot be excluded, while randomized controlled trials are currently lacking. As a result, current recommendations rely mainly on observational evidence, expert consensus, and guideline-based principles, often extrapolated from other cardiovascular populations. Although no formal systematic search or evidence grading was performed, the available literature informing this narrative review is summarized in Table 2, based on a targeted appraisal of studies on exercise training and cardiac rehabilitation after acute aortic dis-section. The table outlines the principal studies underpinning the present discussion, indicating that current evidence is largely derived from small observational cohorts and ex-pert recommendations. While these data support the feasibility and safety of carefully supervised cardiac rehabilitation in selected patients, they also highlight the need for larger, prospectively designed real-world studies.

CR should be regarded as an integral component of post-operative care and initiated as early as clinically appropriate in close collaboration with the surgical team, once clinical stability and hemodynamic compensation are achieved. Therefore, exercise prescriptions should be carefully tailored to each patient’s aortic anatomy, clinical history, and physical capacity [54] and timing should be individualized based on surgical complexity and residual aortic disease. Exercise prescription should be delivered within a coordinated multidisciplinary cardiac rehabilitation model. Although the distribution of responsibilities may vary according to local resources [55], international standards consistently identify exercise prescription as a medical act, with day-to-day supervision delegated to professionals trained in therapeutic exercise. This multidisciplinary model is consistent with cardiac rehabilitation guidelines and implementation studies, which emphasize the importance of role clarity and coordinated team-based care in minimizing risk and optimizing clinical outcomes [56,57,58].

The rehabilitation physician plays a central role in the initial assessment and individualized planning, accounting for surgical sequelae, baseline functional status, and relevant musculoskeletal or neurological comorbidities to appropriately tailor exercise modality and progression [59,60]. In patients with residual aortic disease (e.g., patent false lumen or progressive dilation), exercise intensity should be advanced cautiously with strict blood pressure control and avoidance of high-intensity activities. Surgical complexity and underlying connective tissue disorders further justify a conservative, individualized approach with lower hemodynamic thresholds and long-term surveillance.

Exercise-based CR (ECR) is known to improve blood pressure control, though this benefit is not yet fully confirmed in post-aortic dissection patients. Potential mechanisms include favorable autonomic modulation, reduced sympathetic activity, and improved vascular function, which, together with gains in fitness and weight control [61,62], may enhance antihypertensive effects alongside medical therapy [63]. Strict resting blood pressure control (<130/80 mmHg) remains a key clinical goal and should be regularly monitored. β-blockers are first-line agents to reduce heart rate, systolic pressure, and aortic wall stress, while additional therapies (vasodilators, ACE inhibitors, ARBs, and statins) may support vascular protection when appropriately combined [64]. Fixed-dose combinations may help improve long-term adherence [65].

Rehabilitation may support the recovery of confidence and daily functioning after ATAAD, facilitating return to work, social activities, and intimate life, particularly in younger patients [66]. Given the high rates of anxiety and depression in this population, potentially higher than in other cardiac conditions, psychological support should be a core component of rehabilitation. Although a direct link with outcomes has not been confirmed, depression is known to worsen prognosis in cardiovascular patients, and over half of inactive ATAAD survivors report new-onset depression [67]. Psychological assessment and support should be integral to cardiac rehabilitation, with early and repeated screening using validated tools such as the Patient Health Questionnaire-9 (PHQ-9) for depressive symptoms and the Generalized Anxiety Disorder-7 (GAD-7) for anxiety, or comparable standardized tools, according to the recent Clinical Consensus Statement on mental health and cardiovascular disease [68], and referral to specialized care when needed.

Incorporating mild aerobic exercise may benefit both mental and physical health, while effective weight management requires an integrated approach combining physical activity with nutritional and psychological support [69].

**Table 2:** Comprehensive summary of studies on exercise training and cardiac rehabilitation after acute aortic dissection present in the literature.

Study	Design	Main Outcomes	Key Concepts
Zhou et al. [19]	Observational (retrospective)	Functional capacity	Early low-intensity, BP-controlled; CR feasible.
Delsart et al. [21]	Observational (prospective)	Functional capacity	VO_2_ peak and oxygen pulse predict events; CR feasible.
Norton et al. [22]	Observational (prospective)	Functional capacity; quality-of-life	Post-operative CPET supports risk assessment; CR feasible
Chaddha et al. [23]	Expert opinion	Practical recommendations for exercise	BP-controlled, individualized exercise.
Feng et al. [52]	Scoping review	Evidence for ECR	CR feasible in stable patients; CPET-informed exercise prescription.
Van Iterson et al. [63]	Narrative review	Practical guidance	Low-intensity (<VT1), individualized, supervised exercise.
Schwaab et al. [66]	Consensus recommendations	Practical recommendations for exercise	CR recognized as a key component of functional recovery.
Fuglsang et al. [69]	Observational (retrospective)	Functional capacity; quality-of-life	Resistance training at light intensity combined with aerobic exercise.
Hornsby et al. [70]	Observational (retrospective)	Functional capacity, CPET safe	CPET-guided individualized exercise prescription.

BP, blood pressure; CPET, cardiopulmonary exercise testing; CR, cardiac rehabilitation; ECR, Exercise-based cardiac rehabilitation; VO_2_, oxygen uptake. VT1, first ventilatory threshold.

### 4.1. Aerobic Exercise Training

Following aortic dissection, many patients significantly reduce their physical activity, not due to physical limitations, but fear of recurrence, which may lead to worsened cardiovascular health, elevated resting blood pressure, and psychological distress [67]. However, the currently available data suggest that ECR may be feasible and generally well tolerated in carefully selected, clinically stable patients, with no serious adverse events reported in the published studies [52]. Limited observational data indicate that programs integrating aerobic, resistance, and flexibility training, particularly when individualized and guided by maximal or symptom-limited exercise testing, may be associated with improvements in peak oxygen consumption (VO_2_ peak), exercise capacity, resting blood pressure, and health-related quality of life [69].

Tolerance to ECR appears to be generally favorable, with observational studies reporting increases in peak oxygen consumption in the range of approximately 22–46% and a return to work in more than half of working-age participants [66]. However, evidence remains limited, highlighting the need for standardized, disease-specific rehabilitation protocols. Current guidelines highlight the importance of defining safe exercise thresholds, recommending baseline stress testing with continuous blood pressure monitoring, preferably at low intensity due to high interindividual variability in hemodynamic responses [54,71]. Cardiopulmonary exercise testing (CPET) appears to be safe in carefully selected, clinically stable patients receiving beta-blockers and with well-controlled BP [72]. It may provide useful information on exercise tolerance and functional capacity and could help inform individualized exercise prescription and risk assessment [70]. Notably, in one cohort, peak oxygen pulse and oxygen uptake emerged as independent predictors of future aortic events and major cardiovascular events not directly related to the aorta [21]. CPET also supports the development of tailored exercise prescriptions [52], especially when training zones are defined using ventilatory thresholds (the first and second ventilatory thresholds, VT1 and VT2). A cautious approach involves a minimum 3 min unloaded warm-up to reduce anxiety and stabilize cardiovascular responses. Training should start with several weeks of low-intensity exercise below the first ventilatory threshold (VT1) to ensure safety and tolerability [63]. During testing, systolic blood pressure should be kept below 160 mmHg to minimize risk [66].

During aerobic exercise, systolic blood pressure (SBP) typically rises by 8–12 mmHg per 1 Metabolic Equivalent (MET), regardless of baseline BP, while diastolic pressure changes minimally. In patients with aortic dissection or aneurysm, this SBP rise can place additional stress on the aortic wall, increasing the risk of complications. As a result, high-intensity or strenuous activities (e.g., sprinting, heavy lifting, or resistance training to fatigue) should be avoided. During exercise, aerobic training should be prescribed to limit excessive systolic blood pressure responses, with SBP generally kept below 180 mmHg—or under 160 mmHg in higher-risk patients and during early post-operative phases. However, it should be acknowledged that the proposed SBP limits are based on expert opinion and clinical practice considerations, rather than on validated outcome-based thresholds as no SBP cut-offs have been formally established to predict outcomes post-ATAAD repair. In this context, minimizing excessive blood pressure elevations and limiting BP variability—rather than relying on rigid absolute thresholds—remains a key clinical goal [64]. Accordingly, SBP cut-off values should be interpreted within the broader clinical and hemodynamic context and individualized to patient risk rather than applied rigidly.

**Figure 2:**
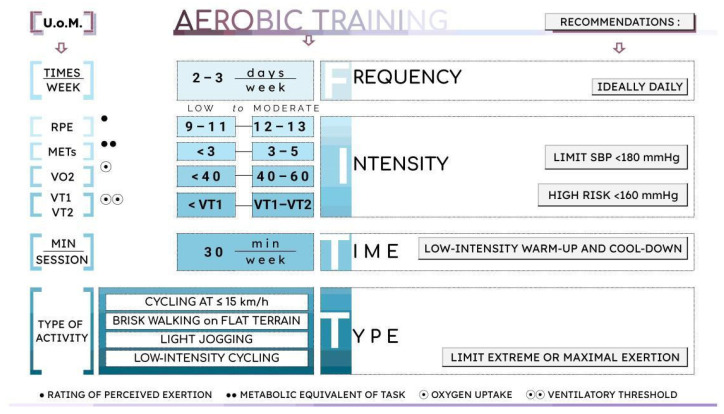
Example of an aerobic training program in acute type A aortic dissection patients surgically resolved based on observational cohorts of clinically stable ATAAD survivors.

Exercise should follow the FITT principle (Frequency, Intensity, Time, Type) [53], with training volume optimized by increasing frequency and duration, thereby maximizing benefits while maintaining low intensity to minimize rises in blood pressure [19]. In observational cohorts of clinically stable ATAAD survivors, structured cardiac rehabilitation programs have generally adopted low-to-moderate exercise intensities, typically in the range of 3–5 METs, corresponding to a Rating of Perceived Exertion (RPE) of 12–13 on the Borg Scale, delivered for approximately 30 min on most days, totaling 150 min weekly with careful blood pressure monitoring during exercise sessions. Suitable activities may include brisk walking, slow jogging, or easy cycling. These approaches have been reported as feasible and well tolerated, although they derive from non-randomized studies and should be interpreted within the context of individualized clinical assessment. Continuous BP monitoring during sessions is essential to ensure safety [67] (Figure 2).

### 4.2. Resistance Exercise Training

The survivors following acute type A aortic dissection tend to be younger and more physically active, rendering physical limitations particularly disruptive to their daily lives [69]. Low-to-moderate-intensity continuous aerobic exercise combined with low-intensity dynamic resistance training may be considered an appropriate approach for cardiac rehabilitation in this population when patients are clinically stable and programs are individualized [54,64].

Resistance training is introduced in stable patients after a careful evaluation performed during the postoperative period. Zhou et al. reported on an early postoperative cardiac rehabilitation program initiated approximately 26.2 ± 17.3 days after surgery, which included continuous aerobic exercise and segmental muscle strengthening five days per week [19]. Importantly, this study found no significant adverse events and concluded that early initiation of cardiac rehabilitation may be safe and feasible in surgically treated ATAAD patients. Nevertheless, no definitive guidelines currently exist regarding the timing of resistance training initiation in this context. Another study confirmed the safety and normal blood pressure response to an afternoon resistance training session [73].

Current literature demonstrates heterogeneity in the types of exercise interventions employed. Only a limited number of studies have explored the benefits of resistance training through calisthenics or isolated muscle contractions involving both upper and lower limbs in conjunction with aerobic training [19,69,74]. To date, there is no evidence supporting the use of resistance training as a standalone intervention in this population, and its isolated effectiveness remains uncertain.

**Figure 3:**
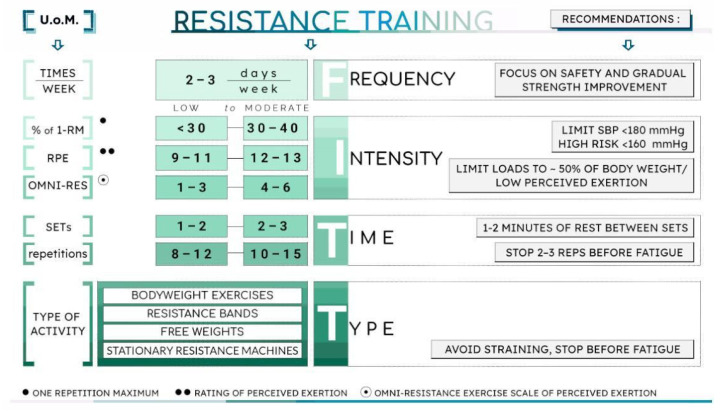
Example of a resistance training program in acute type A aortic dissection patients surgically resolved.

In patients with ATAAD, the primary objective of resistance training should be to enhance functional capacity while minimizing cardiovascular strain particularly by avoiding excessive elevations in blood pressure. However, evidence regarding the safest forms and optimal intensities of resistance training for post-ATAAD patients remains limited [19]. High-intensity static exercises should be avoided, as they are known to produce acute hypertensive responses without yielding long-term reductions in resting blood pressure [75]. Current recommendations suggest that resistance training should be performed at light intensities, typically < 30–40% of the one-repetition maximum (1 RM) [19,69,74]. It has been recommended that ATAAD patients engage in light to moderate resistance training, limiting loads to around 50% of body weight or using equivalent perceived exertion [69]. Subjective tools such as the Borg RPE scale are valuable for monitoring exercise intensity during resistance training. An initial RPE range of 11–14 is recommended, corresponding to perceived effort levels from “light” to “moderately hard” [76]. Similarly, the OMNI-RES scale is a useful alternative, particularly for older adults, with a suggested starting intensity of 3–6, indicating “somewhat light” to “moderately challenging” effort levels [77] (Figure 3).

Some studies incorporated conventional physiotherapy approaches [78], while others included specific modalities such as respiratory muscle training [74]. Despite advancements in surgical and anesthetic techniques, neurological complications including stroke, spinal cord ischemia, and cognitive impairment remain a concern, affecting approximately 18% of patients postoperatively [79]. These individuals may benefit from integrated neurorehabilitation strategies during the long-term recovery phase.

Patients should avoid training to volitional fatigue, terminate sets several repetitions before failure, and refrain from using the Valsalva maneuver, as these factors can lead to excessive increases in blood pressure [80]. For high-risk patients, exercise prescriptions should be tailored according to individual tolerance, with careful attention to maintaining BP within safe limits and preventing large blood pressure fluctuations. Comprehensive assessments such as body composition, peripheral muscle strength, and exercise-induced BP response can support the development of safer and more effective rehabilitation programs. Further research is warranted to better define the safety, feasibility, and long-term benefits of low-to-moderate intensity resistance training in patients recovering from ATAAD surgery.

## 5. Tailored, Multidisciplinary, and Comprehensive Management in Clinical Practice

Expert consensus emphasizes that the primary goal of CR is to improve cardiovascular outcomes through personalized secondary prevention. However, if ECR is not introduced early during follow-up, many patients are never referred, missing a crucial opportunity for recovery. Contemporary guidance highlights that cardiac rehabilitation should not be withheld solely on the basis of high risk, and supports early, carefully individualized CR enrollment following aortic dissection [63]. Programs should be tailored based on medical history, disease severity, medications, functional capacity, surgical outcomes, comorbidities, and patient motivation, to safely guide physical activity and prevent high-risk behaviors such as sudden, intense exertion [53,63,81,82]. Although no specific CR protocols exist for thoracic aortic pathologies, CR may be considered an important component of the recovery process [66].

Patients recovering from aortic dissection often experience reduced aerobic capacity due to chronotropic incompetence and peripheral deconditioning. Fear of exertion and unfamiliarity with structured exercise may lead to misjudging intensity, potentially leading to unsafe exertion levels [63]. Rehabilitation should therefore begin in specialized settings where clinicians can provide structured, progressive training. This approach not only improves physical function but also helps restore patient confidence before patients transition to self-managed activity [72]. A structured care pathway should accompany patients from the immediate postoperative period through all phases of CR: Phase I (inpatient), Phase II (early outpatient), and Phase III (long-term maintenance). Physical exercise should be integrated with core components such as nutritional counseling, psychological support, and ongoing medical supervision to ensure comprehensive and safe recovery.

After surgery for ATAAD, early rehabilitation is crucial to counter hospital-associated disability (HAD), which results from prolonged bed rest, inflammation, and hypermetabolism, all contributing to muscle loss and dysfunction. Although data on HAD in this specific population are limited, early mobilization has been shown to improve functional recovery, reduce ICU and hospital length of stay, and shorten mechanical ventilation time. Rehabilitation may be particularly challenging in patients with complex surgeries or delayed recovery, often linked to longer procedures and higher complication rates [83]. Despite ongoing concerns about blood pressure surges, growing evidence suggests that early initiation of rehabilitation—within the first few days after surgery—may be feasible and safe in carefully selected patients, including some emergency or high-risk cases, when conducted under strict clinical supervision. Early CR has been associated with better functional status at discharge, lower in-hospital mortality, reduced healthcare costs, and shorter hospital stays. These findings align with broader cardiovascular literature and suggest that early CR may represent an important component of recovery following aortic surgery [84]. The immediate postoperative period also marks a key time for patient and family education, including explanations of the surgical procedure, the importance of lifestyle changes, and the role of ongoing rehabilitation.

Upon discharge, patients enter Phase II of CR, typically initiated 3 to 6 weeks after surgery and lasting 8 to 12 weeks. This phase includes supervised, individualized exercise training, based on a detailed assessment of cardiovascular status, surgical outcomes, and functional capacity. Stable patients should regularly undergo individualized maximal exercise testing under optimal medical therapy to tailor safe and effective exercise prescriptions. Since heart rate and blood pressure responses can vary significantly, especially in those taking rate-limiting or antihypertensive medications, regular assessments are essential to refine training regimens over time. β-blockers, a cornerstone of medical therapy in this population, attenuate the chronotropic response to exercise, thereby limiting the reliability of heart rate-based training targets. For this reason, alternative methods such as RPE and, when available, ventilatory thresholds are considered more reliable tools for guiding exercise intensity. A key component of this phase is educating patients on how to apply target heart rate and workload zones specifically during aerobic training. High-risk activities, such as exercises involving intense upper or lower body twisting and high-intensity interval training (HIIT), must be avoided. These precautions, along with continuous professional supervision, help ensure safety, reinforce appropriate exercise behaviors, and optimize rehabilitation outcomes [63].

**Figure 4:**
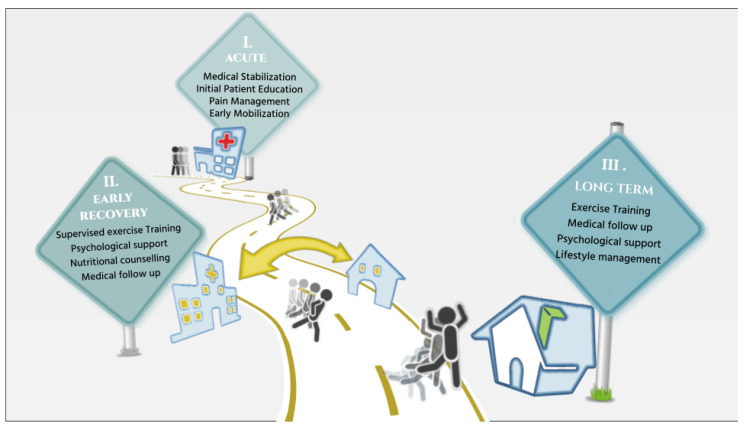
Cardiac rehabilitation program in acute type A aortic dissection patients surgically resolved: tailored assessment and management.

Progression into Phase III marks the start of a long-term maintenance phase, focused on gradually transitioning toward independent, home-based exercise. This is supported by educational and behavioral strategies, and, when available, digital tools such as telerehabilitation and mobile health technologies [85]. Patients should be equipped with the knowledge and skills needed to safely continue exercising, consolidate cardiorespiratory gains, and maintain a healthy lifestyle. Continued clinical supervision is essential, with periodic reassessments to monitor physical condition, blood pressure, lipid profile, and medication adherence. This phase is also key to detecting late complications and sustaining cardiovascular risk reduction. Patients should undergo regular follow ups every 6–12 months, including imaging surveillance (CT or MRI angiography) to monitor aortic dimensions and graft integrity. Although imaging follow-up is routinely recommended, its role in guiding CR decisions is not yet fully standardized. Imaging findings—such as residual dissection, distal aortic dilation, or other high-risk features—should be carefully considered when determining exercise intensity and tolerance thresholds and progression between CR phases. Close collaboration between cardiologists, cardiac surgeons, and rehabilitation teams is essential to ensure that any imaging abnormalities are appropriately integrated into individualized exercise prescriptions and overall post-dissection management. Medical therapy must be consistently optimized, particularly for blood pressure and lipid control, and lifestyle measures—such as diet, physical activity, weight management, and smoking cessation—should be reinforced throughout (Figure 4).

However, despite these promising strategies, specific studies evaluating structured CR pathways in this population are lacking. Although systematic reviews and meta-analyses on CR after thoracic aortic surgery have been proposed [86], robust and targeted evidence remains scarce.

## 6. Conclusions

Cardiac rehabilitation represents a critical yet underutilized opportunity to improve recovery and long-term outcomes in patients after surgical repair of acute type A aortic dissection. When adapted to individual risk profiles and integrated with nutritional counseling, psychological support, and careful hemodynamic monitoring, exercise-based rehabilitation can be delivered safely and effectively. Early mobilization and structured reconditioning help prevent hospital-associated disability, enhance functional status, and promote cardiovascular stability. Despite encouraging preliminary evidence, robust data on long-term benefits and optimal protocols are still lacking. Future research should focus on systematically tracking outcomes in ATAAD survivors through prospective registries and pragmatic clinical trials, aimed at evaluating the safety, feasibility, and long-term benefits of structured cardiac rehabilitation. Developing standardized rehabilitation pathways that consider imaging findings, surgical techniques, and residual aortic pathology will help tailor interventions to individual patient needs. At the same time, integrating behavioral and psychological support can enhance adherence and maximize the benefits of rehabilitation. Together, these efforts are crucial to establish evidence-based guidelines and to position cardiac rehabilitation as a central component of post-dissection care. Future studies are essential to define standardized pathways and to establish rehabilitation as a core component of post-dissection care.

## Figures and Tables

**Table 1:** Proposed aorta-specific checklist for postoperative risk stratification and cardiac rehabilitation referral, timing, and training modification after aortic surgery.

Aorta-specific and surgical factors	Type/complexity of surgery and postoperative course
	Absence of unresolved aortic complications
	Stability of repair on imaging
Hemodynamic and clinical stability	Absence of major postoperative complication
	No uncontrolled arrhythmias, ischemia, HF, infection
	Resting and exertional BP control
Patient-related factors	Cognitive status, adherence, motivation
	Comorbidities (especially connective tissue disorders) and concomitant therapies
	Frailty and deconditioning
Functional and exercise-related assessment	Baseline functional capacity
	Hemodynamic and symptom response to exertion
	Exercise-limiting factors (dyspnea, fatigue, abnormal BP response)
Training principles once CR is initiated	Low-intensity exercise (below VT1)
	Focus on aerobic, light resistance if included
	Avoid Valsalva, isometric strain, and sudden high-intensity exertion
Factors warranting deferral or modification	Uncontrolled hypertension
	Persistent postoperative complications
	Marked frailty or deconditioning
	Inability to ensure close supervision

HF, heart failure; BP, blood pressure; VT1, first ventilatory threshold.

## Data Availability

No new data were created or analyzed in this study. Data sharing is not applicable to this article.

## Abbreviations

The following abbreviations are used in this manuscript:

ACE	Angiotensin-converting enzyme
AD	Aortic dissection
AR	Aortic regurgitation
ARBs	Angiotensin II receptor blockers
ATAAD	Acute type A aortic dissection
BP	Blood pressure
CPET	Cardiopulmonary exercise testing
CR	Cardiac Rehabilitation
CTA	Computed Tomography Angiography
CXR	Chest X-ray
EACTS	The European Association for Cardio-Thoracic Surgery
ECG	Electrocardiogram
ECR	Exercise-based cardiac rehabilitation
FET	Frozen elephant trunk
FITT	Frequency Intensity Time Type
GAD-7	Generalized Anxiety Disorder-7
GERAADA	German Registry of Acute Aortic Dissection Type A
HAD	Hospital-associated disability
HF	Heart failure
HIIT	High-intensity interval training
ICU	Intensive care unit
IRAD	International Registry of Acute Aortic Dissection
MET	Metabolic Equivalent of Task
MRI	Magnetic Resonance Imaging
NMES	Neuromuscular electrical stimulation
OMNI-RES	OMNI Resistance Exercise Scale
PHQ-9	Patient Health Questionnaire-9
RM	Repetition maximum
RPE	Rating of Perceived Exertion
SAV	Supra-aortic vessel
SBP	Systolic blood pressure
STS	The Society of Thoracic Surgeons
TEE	Transesophageal echocardiography
TEM	Type-Entry-Malperfusion
TTE	Transthoracic echocardiography
VO_2_	Oxygen uptake
VT	Ventilatory threshold

## References

[1] Klompas M. (2002). Does this patient have an acute thoracic aortic dissection?. JAMA.

[2] De Bakey M.E., Henly W.S., Cooley D.A., Morris G.C., Crawford E.S., Beall A.C. (1965). SURGICAL MANAGEMENT OF DISSECTING ANEURYSMS OF THE AORTA. J. Thorac. Cardiovasc. Surg..

[3] Grasty M.A., Lawrence K. (2024). Advances and Innovation in Acute Type a Aortic Dissection. J. Clin. Med..

[4] Suzuki Y., DeAnda A. (2021). Revisiting the Death and Autopsy of King George II. AORTA J..

[5] Yin J., Liu F., Wang J., Yuan P., Wang S., Guo W. (2022). Aortic Dissection: Global Epidemiology. Cardiol. Plus.

[6] Obel L.M., Lindholt J.S., Lasota A.N., Jensen H.K., Benhassen L.L., Mørkved A.L., Srinanthalogen R., Christiansen M., Bundgaard H., Liisberg M. (2022). Clinical Characteristics, Incidences, and Mortality Rates for Type A and B Aortic Dissections: A Nationwide Danish Population-Based Cohort Study From 1996 to 2016. Circulation.

[7] Harky A., Singh V.P., Khan D., Sajid M.M., Kermali M., Othman A. (2020). Factors Affecting Outcomes in Acute Type A Aortic Dissection: A Systematic Review. Heart Lung Circ..

[8] Januzzi J.L., Isselbacher E.M., Fattori R., Cooper J.V., Smith D.E., Fang J., Eagle K.A., Mehta R.H., Nienaber C.A., Pape L.A. (2004). Characterizing the Young Patient with Aortic Dissection: Results from the International Registry of Aortic Dissection (IRAD). JACC.

[9] Kamel H., Roman M.J., Pitcher A., Devereux R.B. (2016). Pregnancy and the Risk of Aortic Dissection or Rupture. Circulation.

[10] Wilson-Smith A.R., Eranki A., Muston B., Kamalanathan H., Yung A., Williams M.L., Sahai P., Zi C., Michelena H. (2022). Incidence of Bicuspid Valve Related Aortic Dissection: A Systematic Review and Meta-Analysis. Ann. Cardiothorac. Surg..

[11] Ye Z., Lane C.E., Beachey J.D., Medina-Inojosa J., Galian-Gay L., Dentamaro I., Rodriguez-Palomares J., Calvo-Iglesias F., Paz R.C., Alegret J.M. (2023). Clinical Outcomes in Patients With Bicuspid Aortic Valves and Ascending Aorta ≥50 Mm Under Surveillance. JACC Adv..

[12] Booth K., UK-AS, the UK Aortic Society (2023). Acute Aortic Dissection (AAD)—A Lethal Disease: The Epidemiology, Pathophysiology and Natural History. Br. J. Cardiol..

[13] Jánosi R.A., Buck T., Erbel R. (2009). Mechanism of Coronary Malperfusion Due to Type-A Aortic Dissection. Herz.

[14] Fukui T. (2018). Management of Acute Aortic Dissection and Thoracic Aortic Rupture. J. Intensive Care..

[15] Briggs B., Cline D. (2024). Diagnosing Aortic Dissection: A Review of This Elusive, Lethal Diagnosis. JACEP Open.

[16] Golledge J., Eagle K.A. (2008). Acute Aortic Dissection. Lancet.

[17] Harky A., Chan J., MacCarthy-Ofosu B. (2020). The Future of Stenting in Patients with Type A Aortic Dissection: A Systematic Review. J. Int. Med. Res..

[18] Yamaguchi T., Nakai M., Sumita Y., Miyamoto Y., Matsuda H., Inoue Y., Yoshino H., Okita Y., Minatoya K., Ueda Y. (2020). Current Status of the Management and Outcomes of Acute Aortic Dissection in Japan: Analyses of Nationwide Japanese Registry of All Cardiac and Vascular Diseases-Diagnostic Procedure Combination Data. Eur. Heart J. Acute Cardiovasc. Care.

[19] Zhou N., Fortin G., Balice M., Kovalska O., Cristofini P., Ledru F., Mampuya W.M., Iliou M.-C. (2022). Evolution of Early Postoperative Cardiac Rehabilitation in Patients with Acute Type A Aortic Dissection. J. Clin. Med..

[20] Bossone E., Eagle K.A. (2021). Epidemiology and Management of Aortic Disease: Aortic Aneurysms and Acute Aortic Syndromes. Nat. Rev. Cardiol..

[21] Delsart P., Delahaye C., Devos P., Domanski O., Azzaoui R., Sobocinski J., Juthier F., Vincentelli A., Rousse N., Mugnier A. (2021). Prognostic Value of Aerobic Capacity and Exercise Oxygen Pulse in Postaortic Dissection Patients. Clin. Cardiol..

[22] Norton E.L., Wu K.-H.H., Rubenfire M., Fink S., Sitzmann J., Hobbs R.D., Saberi S., Willer C.J., Yang B., Hornsby W.E. (2022). Cardiorespiratory Fitness After Open Repair for Acute Type A Aortic Dissection—A Prospective Study. Semin. Thorac. Cardiovasc. Surg..

[23] Chaddha A., Eagle K.A., Braverman A.C., Kline-Rogers E., Hirsch A.T., Brook R., Jackson E.A., Woznicki E.M., Housholder-Hughes S., Pitler L. (2015). Exercise and Physical Activity for the Post–Aortic Dissection Patient: The Clinician’s Conundrum. Clin. Cardiol..

[24] Spanos K., Tsilimparis N., Kölbel T. (2018). Exercise after Aortic Dissection: To Run or Not to Run. Eur. J. Vasc. Endovasc. Surg..

[25] Carbone A., Lamberti N., Manfredini R., Trimarchi S., Palladino R., Savriè C., Marra A.M., Ranieri B., Crisci G., Izzo R. (2024). Cardiac Rehabilitation and Acute Aortic Dissection: Understanding and Addressing the Evidence GAP a Systematic Review. Curr. Probl. Cardiol..

[26] Malaisrie S.C., Szeto W.Y., Halas M., Girardi L.N., Coselli J.S., Sundt T.M., Chen E.P., Fischbein M.P., Gleason T.G., Okita Y. (2021). 2021 The American Association for Thoracic Surgery expert consensus document: Surgical treatment of acute type A aortic dissection. J Thorac Cardiovasc Surg..

[27] Benedetti G., Trimarchi G., Palmieri C., Paradossi U., Berti S., Rizza A. (2024). A Case Report of Aortic Intramural Hematoma: From Diagnosis to Endovascular Treatment Guided by Transesophageal Echocardiography. J. Cardiovasc. Echogr..

[28] Vilacosta I., Ferrera C., San Román A. (2024). Acute aortic syndrome. Med. Clin..

[29] Czerny M., Grabenwöger M., Berger T., Aboyans V., Della Corte A., Chen E.P., Desai N.D., Dumfarth J., Elefteriades J.A. (2024). EACTS/STS Guidelines for Diagnosing and Treating Acute and Chronic Syndromes of the Aortic Organ. Ann. Thorac. Surg..

[30] Siani A., Perone F., Costantini P., Rodolfi S., Muscogiuri G., Sironi S., Carriero S., Pavon A.G., van der Bilt I., van Rosendael P. (2022). Aortic regurgitation: A multimodality approach. J. Clin. Ultrasound..

[31] Clark A., Conroy P.D., Feng B., Fadoul M., McMackin K., Lombardi J.V. (2025). Progressive pregnancy-associated aortic dissection requiring eventual replacement of the entire aorta. J. Vasc. Surg. Cases Innov. Tech..

[32] Matei D.C., Robu C., Ciobanu C.G., Antohi E.L., Știru O., Geavlete O.D., Bubenek Ș., Radu R.I., Tinică G., Moldovan H. (2025). Acute Type A Aortic Dissection: Early Mortality Predictors. Cardiol. Rev..

[33] Perone F., Guglielmo M., Coceani M., La Mura L., Dentamaro I., Sabatino J., Gimelli A. (2023). The Role of Multimodality Imaging Approach in Acute Aortic Syndromes: Diagnosis, Complications, and Clinical Management. Diagnostics.

[34] Yao J., Bai T., Yang B., Sun L. (2021). The diagnostic value of D-dimer in acute aortic dissection: A meta-analysis. J. Cardiothorac. Surg..

[35] Von Kodolitsch Y., Nienaber C.A., Dieckmann C., Schwartz A.G., Hofmann T., Brekenfeld C., Nicolas V., Berger J., Meinertz T. (2004). Chest radiography for the diagnosis of acute aortic syndrome. Am. J. Med..

[36] Mazzolai L., Teixido-Tura G., Lanzi S., Boc V., Bossone E., Brodmann M., Bura-Rivière A., De Backer J., Deglise S., Della Corte A. (2024). ESC Scientific Document Group. 2024 ESC Guidelines for the management of peripheral arterial and aortic diseases. Eur. Heart J..

[37] Evangelista A., Avegliano G., Aguilar R., Cuellar H., Igual A., González-Alujas T., Rodríguez-Palomares J., Mahia P., García-Dorado D. (2010). Impact of contrast-enhanced echocardiography on the diagnostic algorithm of acute aortic dissection. Eur. Heart J..

[38] Teurneau-Hermansson K., Ede J., Larsson M., Linton G., von Rosen D., Sjögren J., Wierup P., Nozohoor S., Zindovic I. (2024). Mortality after non-surgically treated acute type A aortic dissection is higher than previously reported. Eur. J. Cardiothorac. Surg..

[39] Trimarchi G., Benedetti G., Palmieri C., Rizza A. (2023). A Case of Type B Aortic Dissection: The Role of Transesophageal Ultrasound Guidance in Thoracic Endovascular Aortic Repair. J. Cardiovasc. Echogr..

[40] Rizza A., Trimarchi G., Di Sibio S., Bastiani L., Murzi M., Palmieri C., Foffa I., Berti S. (2023). Preliminary Outcomes of Zone 2 Thoracic Endovascular Aortic Repair Using Castor Single-Branched Stent Grafts: A Single-Center Experience. J. Clin. Med..

[41] Hagan P.G., Nienaber C.A., Isselbacher E.M., Bruckman D., Karavite D.J., Russman P.L., Evangelista A., Fattori R., Suzuki T., Oh J.K. (2000). The International Registry of Acute Aortic Dissection (IRAD): New insights into an old disease. JAMA.

[42] Rylski B., Schilling O., Czerny M. (2023). Acute aortic dissection: Evidence, uncertainties, and future therapies. Eur. Heart J..

[43] De Paulis S., Arlotta G., Calabrese M., Corsi F., Taccheri T., Antoniucci M.E., Martinelli L., Bevilacqua F., Tinelli G., Cavaliere F. (2022). Postoperative Intensive Care Management of Aortic Repair. J. Pers. Med..

[44] Meredith E.L., Masani N.D. (2009). Echocardiography in the emergency assessment of acute aortic syndromes. Eur. J. Echocardiogr..

[45] McDonagh T.A., Metra M., Adamo M., Gardner R.S., Baumbach A., Böhm M., Burri H., Butler J., Čelutkienė J., Chioncel O. (2021). 2021 ESC Guidelines for the diagnosis and treatment of acute and chronic heart failure. Eur. Heart J..

[46] Bauer T.M., Yaser J.M., Daramola T., Mansour A.I., Ailawadi G., Pagani F.D., Theurer P., Likosky D.S., Keteyian S.J., Thompson M.P. (2023). Cardiac Rehabilitation Reduces 2-Year Mortality After Coronary Artery Bypass Grafting. Ann. Thorac. Surg..

[47] Cassina T., Putzu A., Santambrogio L., Villa M., Licker M.J. (2016). Hemodynamic challenge to early mobilization after cardiac surgery: A pilot study. Ann. Card. Anaesth..

[48] Kourek C., Dimopoulos S. (2024). Cardiac rehabilitation after cardiac surgery: An important underutilized treatment strategy. World J. Cardiol..

[49] Chen B., Xie G., Lin Y., Chen L., Lin Z., You X., Xie X., Dong D., Zheng X., Li D. (2021). A systematic review and meta-analysis of the effects of early mobilization therapy in patients after cardiac surgery. Medicine.

[50] Kourek C., Nanas S., Kotanidou A., Raidou V., Dimopoulou M., Adamopoulos S., Karabinis A., Dimopoulos S. (2022). Modalities of Exercise Training in Patients with Extracorporeal Membrane Oxygenation Support. J. Cardiovasc. Dev. Dis..

[51] Malone D., Ridgeway K., Nordon-Craft A., Moss P., Schenkman M., Moss M. (2015). Physical Therapist Practice in the Intensive Care Unit: Results of a National Survey. Phys. Ther..

[52] Feng D., Ke J., Huang S., Lang X. (2021). A scoping review of exercise-based cardiac rehabilitation for patients with aortic dissection. Rev. Cardiovasc. Med..

[53] Ambrosetti M., Abreu A., Corrà U., Davos C.H., Hansen D., Frederix I., Iliou M.C., Pedretti R.F.E., Schmid J.P., Vigorito C. (2021). Secondary prevention through comprehensive cardiovascular rehabilitation: From knowledge to implementation. 2020 update. A position paper from the Secondary Prevention and Rehabilitation Section of the European Association of Preventive Cardiology. Eur. J. Prev. Cardiol..

[54] Pelliccia A., Sharma S., Gati S., Bäck M., Börjesson M., Caselli S., Collet J.P., Corrado D., Drezner J.A., Halle M. (2021). 2020 ESC Guidelines on sports cardiology and exercise in patients with cardiovascular disease. Eur. Heart J..

[55] Supervia M., Turk-Adawi K., Lopez-Jimenez F., Pesah E., Ding R., Britto R.R., Bjarnason-Wehrens B., Derman W., Abreu A., Babu A.S. (2019). Nature of Cardiac Rehabilitation Around the Globe. EClinicalMedicine.

[56] Thomas R.J. (2024). Cardiac Rehabilitation—Challenges, Advances, and the Road Ahead. N. Engl. J. Med..

[57] Brown T.M., Pack Q.R., Aberegg E., Brewer L.C., Ford Y.R., Forman D.E., Gathright E.C., Khadanga S., Ozemek C., Thomas R.J. (2024). Core Components of Cardiac Rehabilitation Programs: 2024 Update: A Scientific Statement from the American Heart Association and the American Association of Cardiovascular and Pulmonary Rehabilitation. Circulation.

[58] King M., Bittner V., Josephson R., Lui K., Thomas R.J., Williams M.A. (2012). Medical director responsibilities for outpatient cardiac rehabilitation/secondary prevention programs: 2012 update: A statement for health care professionals from the American Association of Cardiovascular and Pulmonary Rehabilitation and the American Heart Association. Circulation.

[59] Nowak J., Listewnik M., Rył A., Pacholewicz J., Rotter I. (2025). Rehabilitation Progress in Patients Following Surgery for Acute Stanford Type A Aortic Dissection Extending Beyond the Ascending Aorta. J. Clin. Med..

[60] Heybati K., Ochal D., Poudel K., Zuberi E., Deng J., Taylor B.J., Dineen E.H., Brahmbhatt P.M., Daugherty D.H., Farres S. (2025). Patient Characteristics and Outcomes of Cardiac Rehabilitation Following Thoracic Aortic Dissection Surgery: A Multicenter Retrospective Study. J. Cardiopulm. Rehabil. Prev..

[61] Popiolek-Kalisz J., Mazur M., Perone F. (2025). The Role of Dietary Education in Cardiac Rehabilitation. Nutrients.

[62] Perone F., Spadafora L., Pratesi A., Nicolaio G., Pala B., Franco G., Ruzzolini M., Ambrosetti M. (2024). Obesity and cardiovascular disease: Risk assessment, physical activity, and management of complications. Int. J. Cardiol. Cardiovasc. Risk Prev..

[63] Van Iterson E.H., Laffin L.J., Svensson L.G., Cho L. (2022). Individualized exercise prescription and cardiac rehabilitation following a spontaneous coronary artery dissection or aortic dissection. Eur. Heart J. Open..

[64] Zhou N., Mampuya W.M., Iliou M.C. (2022). Is Exercise Blood Pressure Putting the Brake on Exercise Rehabilitation after Acute Type A Aortic Dissection Surgery?. J. Clin. Med..

[65] Perrone V., Veronesi C., Gambera M., Nati G., Perone F., Tagliabue P.F., Degli Esposti L., Volpe M. (2019). Treatment with Free Triple Combination Therapy of Atorvastatin, Perindopril, Amlodipine in Hypertensive Patients: A Real-World Population Study in Italy. High. Blood Press. Cardiovasc. Prev..

[66] Schwaab B., Rauch B., Völler H., Benzer W., Schmid J.P. (2022). Beyond randomised studies: Recommendations for cardiac rehabilitation following repair of thoracic aortic aneurysm or dissection. Eur. J. Prev. Cardiol..

[67] Chaddha A., Kline-Rogers E., Braverman A.C., Erickson S.R., Jackson E.A., Franklin B.A., Woznicki E.M., Jabara J.T., Montgomery D.G., Eagle K.A. (2015). Survivors of Aortic Dissection: Activity, Mental Health, and Sexual Function. Clin. Cardiol..

[68] Bueno H., Deaton C., Farrero M., Forsyth F., Braunschweig F., Buccheri S., Dragan S., Gevaert S., Held C., Kurpas D. (2025). ESC Scientific Document Group. 2025 ESC Clinical Consensus Statement on mental health and cardiovascular disease: Developed under the auspices of the ESC Clinical Practice Guidelines Committee. Eur. Heart J..

[69] Fuglsang S., Heiberg J., Hjortdal V.E., Laustsen S. (2017). Exercise-based cardiac rehabilitation in surgically treated type-A aortic dissection patients. Scand. Cardiovasc. J..

[70] Hornsby W.E., Norton E.L., Fink S., Saberi S., Wu X., McGowan C.L., Brook R.D., Jones L.W., Willer C.J., Patel H.J. (2020). Cardiopulmonary Exercise Testing Following Open Repair for a Proximal Thoracic Aortic Aneurysm or Dissection. J. Cardiopulm. Rehabil. Prev..

[71] Chaddha A., Kline-Rogers E., Woznicki E.M., Brook R., Housholder-Hughes S., Braverman A.C., Pitler L., Hirsch A.T., Eagle K.A. (2014). Cardiology patient page. Activity recommendations for postaortic dissection patients. Circulation.

[72] Delsart P., Maldonado-Kauffmann P., Bic M., Boudghene-Stambouli F., Sobocinski J., Juthier F., Domanski O., Coisne A., Azzaoui R., Rousse N. (2016). Post aortic dissection: Gap between activity recommendation and real life patients aerobic capacities. Int. J. Cardiol..

[73] Li J., Boyd A., Huang M., Berookhim J., Prakash S.K. (2022). Safety of exercise for adults with thoracic aortic aneurysms and dissections. Front. Sports Act Living.

[74] Corone S., Iliou M.C., Pierre B., Feige J.M., Odjinkem D., Farrokhi T., Bechraoui F., Hardy S., Meurin P. (2009). Cardiac Rehabilitation working Group of the French Society of Cardiology. French registry of cases of type I acute aortic dissection admitted to a cardiac rehabilitation center after surgery. Eur. J. Cardiovasc. Prev. Rehabil..

[75] Niewiadomski W., Pilis W., Laskowska D., Gąsiorowska A., Cybulski G., Strasz A. (2012). Effects of a brief Valsalva manoeuvre on hemodynamic response to strength exercises. Clin. Physiol. Funct. Imaging.

[76] Borg G.A. (1982). Psychophysical bases of perceived exertion. Med. Sci. Sports Exerc..

[77] Gearhart R.F., Lagally K.M., Riechman S.E., Andrews R.D., Robertson R.J. (2009). Strength tracking using the OMNI resistance exercise scale in older men and women. J. Strength. Cond. Res..

[78] Tashima Y., Toyoshima Y., Chiba K., Nakamura N., Adachi K., Inoue Y., Yamaguchi A. (2021). Physical activities and surgical outcomes in elderly patients with acute type A aortic dissection. J. Card. Surg..

[79] Chiappini B., Schepens M., Tan E., Dell’Amore A., Morshuis W., Dossche K., Bergonzini M., Camurri N., Reggiani L.B., Marinelli G. (2005). Early and late outcomes of acute type A aortic dissection: Analysis of risk factors in 487 consecutive patients. Eur. Heart J..

[80] Thijssen C.G.E., Bons L.R., Gökalp A.L., Van Kimmenade R.R.J., Mokhles M.M., Pelliccia A., Takkenberg J.J.M., Roos-Hesselink J.W. (2019). Exercise and sports participation in patients with thoracic aortic disease: A review. Expert. Rev. Cardiovasc. Ther..

[81] Adam C.A., Erskine J., Akinci B., Kambic T., Conte E., Manno G., Halasz G., Sileikiene V., Fogacci F., Perone F. (2025). Exercise Training and Cardiac Rehabilitation in Patients After Percutaneous Coronary Intervention: Comprehensive Assessment and Prescription. J. Clin. Med..

[82] Ciuca-Pană M.A., Boulmpou A., Ileri C., Manzi G., Golino M., Ostojic M., Galimzhanov A., Busnatu S., Mega S., Perone F. (2025). Chronic Heart Failure and Coronary Artery Disease: Pharmacological Treatment and Cardiac Rehabilitation. Medicina.

[83] Hirakawa K., Nakayama A., Saitoh M., Hori K., Shimokawa T., Iwakura T., Haraguchi G., Isobe M. (2022). Factors Related to Hospitalisation-Associated Disability in Patients after Surgery for Acute Type A Aortic Dissection: A Retrospective Study. Int. J. Environ. Res. Public. Health..

[84] Nakamura K., Ohbe H., Uda K., Matsui H., Yasunaga H. (2022). Effectiveness of early rehabilitation following aortic surgery: A nationwide inpatient database study. Gen. Thorac. Cardiovasc. Surg..

[85] Scherrenberg M., Falter M., Abreu A., Aktaa S., Busnatu S., Casado-Arroyo R., Dendale P., Dilaveris P., Locati E.T., Marques-Sule E. (2025). Standards for cardiac telerehabilitation. Eur. Heart J..

[86] Koenders N., van Zetten H., Smulders M., Verra M.L., van Kimmenade R.R.J., van Brakel T., Eijsvogels T.M.H., Smith T. (2023). Outcomes after cardiac rehabilitation in patients following repair of thoracic aortic aneurysm or dissection: A protocol for a systematic review and meta-analysis. Syst. Rev..

